# REVEILLE Transcription Factors Contribute to the Nighttime Accumulation of Anthocyanins in ‘Red Zaosu’ (*Pyrus Bretschneideri* Rehd.) Pear Fruit Skin

**DOI:** 10.3390/ijms21051634

**Published:** 2020-02-27

**Authors:** Xieyu Li, Ting Wu, Hanting Liu, Rui Zhai, Yao Wen, Qianrong Shi, Chengquan Yang, Zhigang Wang, Fengwang Ma, Lingfei Xu

**Affiliations:** 1 College of Horticulture, Northwest A&F University, Taicheng Road NO.3, Yangling 712100, China; 2 State Key Laboratory of Crop Stress Biology for Arid Areas, Northwest A&F University, Taicheng Road NO.3, Yangling 712100, China

**Keywords:** anthocyanin accumulation, night, red pear fruit, *REVEILLEs*, transient assays

## Abstract

Anthocyanin biosynthesis exhibits a rhythmic oscillation pattern in some plants. To investigate the correlation between the oscillatory regulatory network and anthocyanin biosynthesis in pear, the anthocyanin accumulation and the expression patterns of anthocyanin late biosynthetic genes (*ALBG*s) were investigated in fruit skin of ‘Red Zaosu’ (*Pyrus bretschneideri* Rehd.). The anthocyanin accumulated mainly during the night over three continuous days in the fruit skin, and the *ALBG*s’ expression patterns in ‘Red Zaosu’ fruit skin were oscillatory. However, the expression levels of typical anthocyanin-related transcription factors did not follow this pattern. Here, we found that the expression patterns of four *PbREVEILLE*s (*PbRVE*s), members of a class of atypical anthocyanin-regulated *MYB*s, were consistent with those of *ALBG*s in ‘Red Zaosu’ fruit skin over three continuous days. Additionally, transient expression assays indicated that the four *PbRVE*s promoted anthocyanin biosynthesis by regulating the expression of the anthocyanin biosynthetic genes encoding dihydroflavonol-4-reductase (DFR) and anthocyanidin synthase (ANS) in red pear fruit skin, which was verified using a dual-luciferase reporter assay. Moreover, a yeast one-hybrid assay indicated that *PbRVE1a*, *1b* and *7* directly bound to *PbDFR* and *PbANS* promoters. Thus, *PbRVE*s promote anthocyanin accumulation at night by up-regulating the expression levels of *PbDFR* and *PbANS* in ‘Red Zaosu’ fruit skin.

## 1. Introduction

Anthocyanins are a group of water-soluble flavonoid metabolites that exist widely in plants [1]. Anthocyanins play various roles in plant growth and development [2,3,4,5]. In plants, the antioxidant capacities of anthocyanins rely on the extent of B-ring hydroxylation, the type and degree of acylation and glycosylation [2,3,4]. Anthocyanins play important roles in promoting plant reproduction by transmitting bright colors to pollinators and seed spreaders [2,5]. Additionally, anthocyanins have human health-related benefits [6,7,8,9]. Therefore, investigating anthocyanin accumulation in fruit is worthwhile.

In *A. thaliana*, the anthocyanin biosynthetic pathway is regulated by multiple enzymes, including early and late biosynthetic genes. The early biosynthetic genes include chalcone synthase, chalcone isomerase, flavanone-3-hydroxylase and flavonoid 3′-hydroxylase, while the late biosynthetic genes include dihydroflavonol 4-reductase (DFR), anthocyanidin synthase (ANS) and UDP-glucoside: flavonoid glucosyltransferase (UFGT) [1,10,11]. Many transcription factors (TFs), such as MYBs, bHLHs and WD40, also participate in the anthocyanin biosynthetic pathway. R2R3–MYB proteins interact with bHLH proteins, such as TRANSPARENT TESTA8, GLABRA3 and ENHANCER OF GLABRA3, and form MYB–bHLH–WDR (MBW) complexes with the WD40 protein TRANSPARENT TESTA GLABRA1 [12,13,14]. MBW complexes participate in developmental regulation, physiological regulation, trichome formation, seed coat differentiation and the flavonoid biosynthetic pathway in plants [11]. This ternary, complex protein regulates anthocyanin biosynthesis in most plants [12,13,14,15]. In the dicot *A. thaliana*, the late biosynthetic genes are activated by the MBW complex [12,13,14]. In the monocot maize, the anthocyanin biosynthetic genes are activated as a unit by the MBW complex [15]. Additionally, some typical *R2R3–MYB* and *bHLH* TFs act as anthocyanin-related activators in various plants, such as *A. thaliana*, apple, grape and pear [1,16,17,18,19,20,21,22]. In *A. thaliana*, the MYB TFs *PAP1 (AtMYB75)*, *PAP2 (AtMYB90)*, *AtMYB113* and *AtMYB114* have been described as positive regulators in anthocyanin accumulation [1,16,17]. In apple, anthocyanin biosynthesis is promoted by *MdbHLH3*, *MdMYB88* and *MdMYB124* under low night-temperature conditions [18,19]. *MYB10* and *MYB10b* function as activate regulators in the anthocyanin biosynthetic pathway of pear fruit [20,21,22]. 

In plants, the circadian system coordinates physiology and metabolism with the most appropriate or favorable time of day or season [23]. Plants can save energy and resources by regulating reaction times; therefore, the circadian system is crucial to the health and survival of plants. The anthocyanin biosynthetic pattern has diurnal oscillations in some plants. In *A. thaliana*, the expression levels of phenylalanine ammonia lyase, chalone synthase, chalcone isomerase, and *DFR* are regulated by a circadian clock [24]. *MYBL2* and *MYBD* are regulated by circadian rhythms and involved in the anthocyanin biosynthetic pathway of *A. thaliana* [25,26]. Single MYB TFs, named *REVEILLEs* (*LHY-CCA1-LIKE*) act as important regulators of circadian clockwork [27]. Moreover, in *A. thaliana*, an atypical *MYB* (single MYB TF) named *AtRVE8* regulates anthocyanin biosynthesis by binding directly to the promoters of anthocyanin structural genes, and *AtRVE8* also regulates the expression levels of genes in response to diurnal oscillations [27]. However, the rhythmic regulation of anthocyanin biosynthesis has remains largely unknown in most fruit.

In this study, we isolated four *RVE*s of ‘Red Zaosu’ pear (*Pyrus bretschneideri* Rehd.) because of the linkage between their expression patterns and nighttime increases in anthocyanin biosynthesis. Moreover, we found that the expression levels of the four *RVE*s exhibited rhythmic oscillation patterns in ‘Red Zaosu’ fruit skin. Then, we investigated the functions of the *PbRVE*s in anthocyanin accumulation in pear fruit skin. This study confirmed that *PbRVE*s promote anthocyanin accumulation by up-regulating the expression levels of *PbDFR* and *PbANS* in pear fruit skin.

## 2. Results

### 2.1. The Anthocyanin Content Oscillated Diurnally and Mainly Increased over Night in ‘Red Zaosu’ Fruit Skin

**Figure 1:**
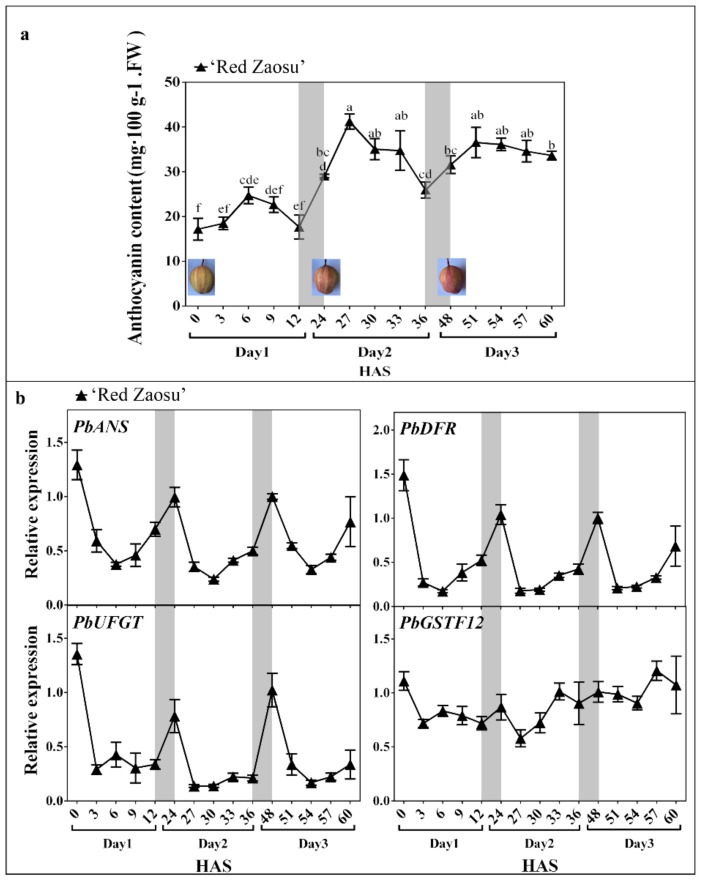
Daily change patterns in anthocyanin content, and the expression patterns of anthocyanin late biosynthetic genes (*ALBG*s) and a typical anthocyanin transport gene, *PbGSTF12*, in ‘Red Zaosu’ pear fruit skin. (**a**) The anthocyanin content in the fruit skin of ‘Red Zaosu’ over three continuous days. Images show the ‘Red Zaosu’ phenotypes at three time points over the three continuous days. (**b**) The expression patterns of *ALBG*s and *PbGSTF12* in ‘Red Zaosu’ fruit skin over three continuous days. The gray boxes indicate nighttime. The NCBI accession numbers of the *ALBG*s and *PbGSTF12* are listed in Supplementary Table S1. HAS: hours after sunrise of day 1. Error bars represent the standard errors (SEs) of the means (*n* = 3). Data in (a) was determined using a one-way analysis of variance (*p* < 0.05); the significant differences are indicated by different lowercase letters.

To determine whether anthocyanin accumulates during the daytime or nighttime in red-skinned pear fruit, the anthocyanin content of ‘Red Zaosu’ fruit skin was measured during the daytime (from sunrise to sunset). Moreover, to accurately observe the anthocyanin accumulation in pear fruit skin during a short period of time, color-faded bagged fruit were used because of the low background level of anthocyanin. Color-faded fruit of ‘Red Zaosu’ were exposed to sunlight to re-accumulate anthocyanin over three continuous days. The anthocyanin content in ‘Red Zaosu’ fruit skin rhythmically increased after sunrise and then decreased from noon to sunset (Figure 1a). However, the anthocyanin content between sunrise and sunset was not significantly different during the course of a day. This phenomenon was also observed in other three red pear varieties (Supplementary Figure S1a). However, a significant increase in the anthocyanin content of ‘Red Zaosu’ fruit skin was detected from sunset to the next sunrise over three continuous days (Figure 1a). Thus, the anthocyanin content in the skin of red pear fruit mainly accumulated during the night.

We further investigated the expression patterns of the anthocyanin late biosynthetic genes (*ALBG*s), including *PbANS*, *PbDFR* and *PbUFGT*, and a typical anthocyanin transporter gene, *PbGSTF12*, in the fruit skin of ‘Red Zaosu’ over three continuous days. The expression levels of the *ALBG*s in the fruit skin of ‘Red Zaosu’ rhythmically decreased from sunrise to sunset and then increased until the next sunrise over the three continuous days (Figure 1b). The significant nighttime increase in expression was observed for each of the *ALBG*s, but not for *PbGSTF12*, over the three continuous days (Figure 1b). Thus, the accumulation of anthocyanin in ‘Red Zaosu’ fruit skin occurred mainly during the night rather than during the day.

### 2.2. The Expression Patterns of Candidate REVEILLE (RVE) TFs Correlated with the Nighttime Increase in the Anthocyanin Level in ‘Red Zaosu’ Fruit Skin

**Figure 2:**
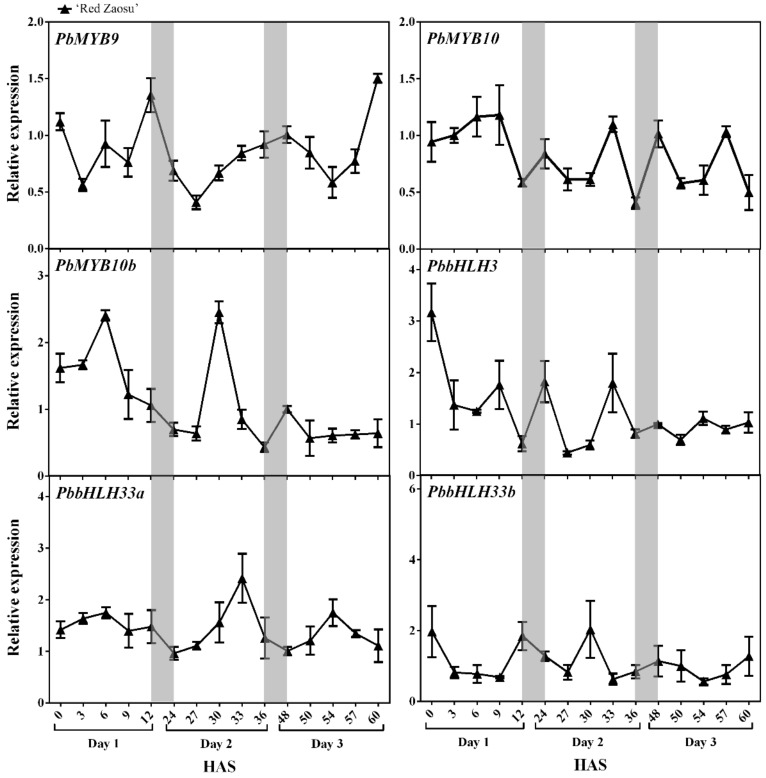
The expression patterns of typical anthocyanin-related transcription factors in ‘Red Zaosu’ pear fruit skin. The expression patterns of *PbMYB9*, *PbMYB10*, *PbMYB10b*, *PbbHLH3*, *PbbHLH33a* and *PbbHLH33b* in ‘Red Zaosu’ fruit skin over three continuous days. The gray boxes indicate nighttime. HAS: hours after sunrise of day 1. The NCBI accession numbers of the typical transcription factors are listed in Supplementary Table S1. Error bars represent SEs of the means (*n* = 3).

To identify the candidate regulators of the nighttime increases in anthocyanin in ‘Red Zaosu’ pear fruit skin, the expression levels of typical anthocyanin-related TFs, including *PbMYB9*, *PbMYB10*, *PbMYB10b*, *PbbHLH3*, *PbbHLH33a* and *PbbHLH33b*, were initially investigated in the fruit skin of ‘Red Zaosu’ over three continuous days (Figure 2). Surprisingly, none of these TFs showed an expression pattern similar to that of the *ALBG*s (Figure 2). Therefore, we further focused on the *RVE*s because of the linkage between their expression patterns and anthocyanin biosynthesis [27].

**Figure 3:**
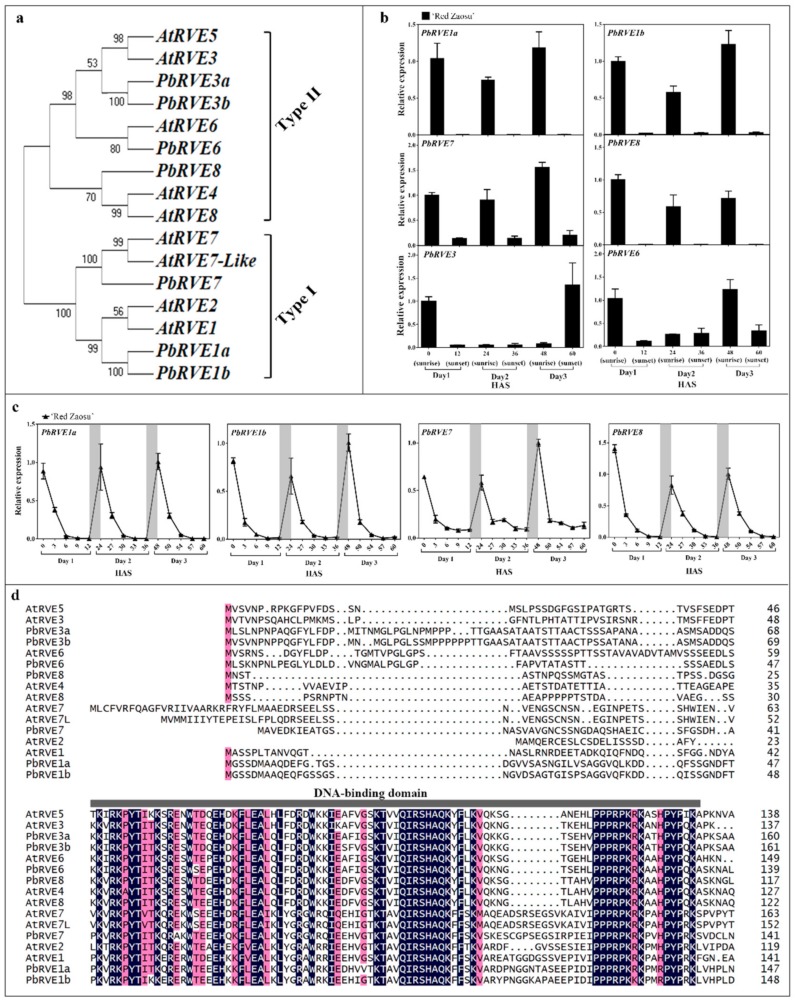
Phylogenetic analysis of *REVEILLEs* (*RVE*s) and the expression patterns of *PbRVE*s in ‘Red Zaosu’ pear fruit skin. (**a**) Phylogenetic analysis of RVE proteins from pear and *A. thaliana*. The type I proteins are AtRVE7, AtRVE7-Like, PbRVE7, AtRVE1, AtRVE2, PbRVE1a and PbRVE1b; the type II proteins are AtRVE3, AtRVE5, PbRVE3a, PbRVE3b, AtRVE6, PbRVE6, PbRVE8, AtRVE4 and AtRVE8. (**b**) The expression patterns of *PbRVE*s in ‘Red Zaosu’ fruit skin at sunrise and sunset over three continuous days. HAS: hours after sunrise of day 1. The actual transcript abundance data of *PbRVEs* are listed in Supplementary Table S3. (**c**) The expression patterns of *PbRVE1a*, *1b*, *7* and *8* in ‘Red Zaosu’ fruit skin over three continuous days. The gray boxes indicate nighttime. (**d**) Multiple sequence alignment of RVE proteins. RVE proteins were aligned using ClustalW. The DNA-binding domain is indicated by a horizontal gray bar. The phylogenetic analysis was constructed with the Neighbor-joining method (1000 replications bootstrap test and JTT model distribution) using MEGA 7.0. The protein sequences of the RVEs were obtained from the pear genome. The gene accession numbers are listed in Supplementary Table S1. Error bars represent SEs of the means (*n* = 3).

Seven selected pear *RVE*s were isolated from the Chinese pear genome [22] (http://peargenome.njau.edu.cn/, March 1, 2018). To analyze the relationship between PbRVE and AtRVE proteins, a phylogenetic tree was constructed using the Neighbor-Joining method (Figure 3a). These PbRVE proteins were classified into two subgroups. The PbRVE1s and PbRVE7 clustered into type I, while the PbRVE3s, PbRVE6 and PbRVE8 clustered into type II (Figure 3a). 

To identify which *PbRVE*s participated in anthocyanin accumulation during the night, we analyzed the expression patterns of *PbRVE*s in the skin of ‘Red Zaosu’ fruit at sunrise and sunset over the three continuous days. The expression levels of *PbRVE1a*, *1b*, *7* and *8*, but not *PbRVE3a*, *3b* and *6*, significantly increased during the nighttime in skin of ‘Red Zaosu’ fruit (Figure 3b). Therefore, the expression levels of *PbRVE1a*, *1b*, *7* and *8* in the skin of ‘Red Zaosu’ fruit were further investigated over three continuous days. *PbRVE1a*, *1b*, *7* and *8* expression levels peaked at dawn and then decreased until sunset during each day (Figure 3c). Furthermore, this expression pattern was also found in the skins of other red pear cultivars (Supplementary Figure S1b). Moreover, at sunrise and sunset of each of three continuous days, significant correlations were observed between *PbRVE* and *ALBG* expression levels in ‘Red Zaosu’ fruit skin, while the expression levels of *PbMYB9*, *PbMYB10*, *PbMYB10b* and *PbbHLH33a* were only slightly correlated with those of *PbANS*, *PbDFR* and *PbUFGT* (Table 1). A multiple-alignment showed that all the candidate *PbRVE*s and *AtRVE*s contained the conserved SHAQK[Y/F]F motif in the DNA-binding domains of their *N*-terminal regions (Figure 3d). Thus, *PbRVE1a*, *1b*, *7* and *8* were selected as candidate genes because of the high correlations between their expression and the expression of *ALBG*s in pear fruit skin. 

### 2.3. Overexpression of PbRVEs in ‘Zaosu’ Pear Fruit Promoted Anthocyanin Accumulation

**Figure 4:**
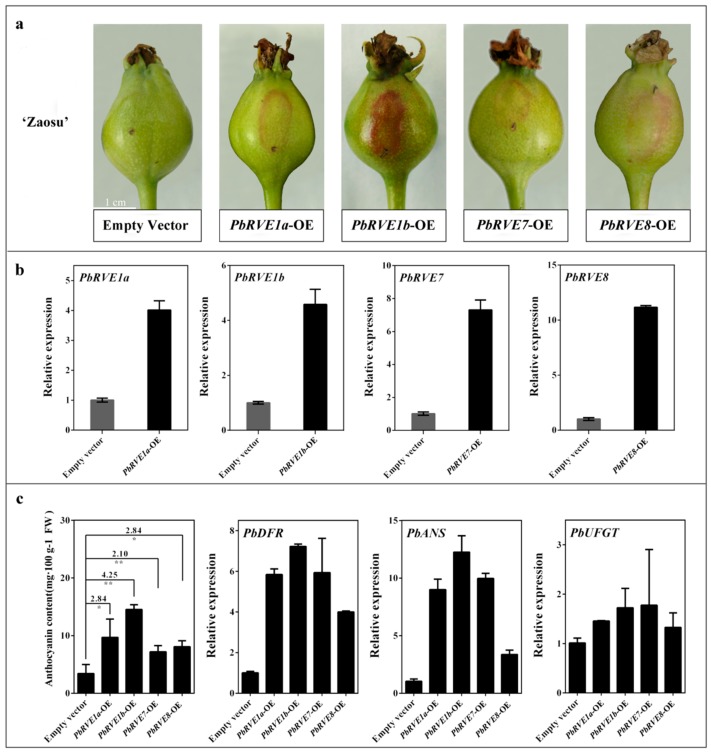
Anthocyanin biosynthetic patterns in *RVE*-overexpression. (**a**) Phenotypes of ‘Zaosu’ fruitlet skins transiently overexpressing *PbRVE1a*, *1b*, *7* and *8*. (**b**) The overexpression levels of *PbRVE1a*, *1b*, *7* and *8* in ‘Zaosu’ fruitlet skins. (**c**) The anthocyanin contents and the expression patterns of *ALBG*s in ‘Zaosu’ fruitlet skins which overexpressing *PbRVEs*. The number means the ratio of anthocyanin content in ‘Zaosu’ fruitlets skin overexpressing *PbRVE*s compared with the empty vector. OE: overexpression. The significant differences are based on comparisons with the empty vector. Error bars indicate the SEs of the means (*n* = 3). Data in (c) were analyzed using Student’s *t*-test: * *p* < 0.05, ** *p* < 0.01.

To investigate the bio-functions of *PbRVE*s in anthocyanin regulation, these candidates were transiently overexpressed using agrobacterium-infiltration of the skin of ‘Zaosu’ fruitlets. The validity of the ‘Zaosu’ fruitlets’ infection was validated by monitoring the β-glucuronidase gene (GUS) signal (Supplementary Figure S2). The transient overexpression of *PbRVE1a*, *1b*, *7* and *8* independently in ‘Zaosu’ fruitlet skins increased the anthocyanin accumulation (Figure 4). However, the promotive efficiency among these *PbRVE*s varied (Figure 4a,b). Consequently, the overexpression of *PbRVE1b* resulted in an intense pigmentation of the pear fruitlet skins (4.25 times darker than controls), whereas lighter pigmentation was observed when *PbRVE1a*, *7*, and *8* were independently overexpressed in pear fruitlet skins (from 2.10 to 2.84 times darker than controls) (Figure 4c). Additionally, the virus-induced gene silencing (VIGS) of *PbRVE1a*, *1b*, *7* and *8* independently in ‘Palacer’ (*P. communis* L.) fruitlet skins decreased the anthocyanin accumulation (Supplementary Figure S3). The expression levels of *PbDFR* and *PbANS* significantly increased in *PbRVEs*-overexpression (OE) ‘Zaosu’ fruitlet skins, especially *PbRVE1b*-OE ‘Zaosu’ fruitlet skins, but the expression level of *PbUFGT* was not affected (Figure 4c). Thus, *PbRVE*s promote the expression levels of *PbDFR* and *PbANS* to increase the anthocyanin accumulation in ‘Zaosu’ pear fruit. 

### 2.4. PbRVE1a, 1b, 7 and 8 Promoted Anthocyanin Accumulation by Activating the Promoters of ALBGs in ‘Red Zaosu’ Pear Fruit

**Figure 5:**
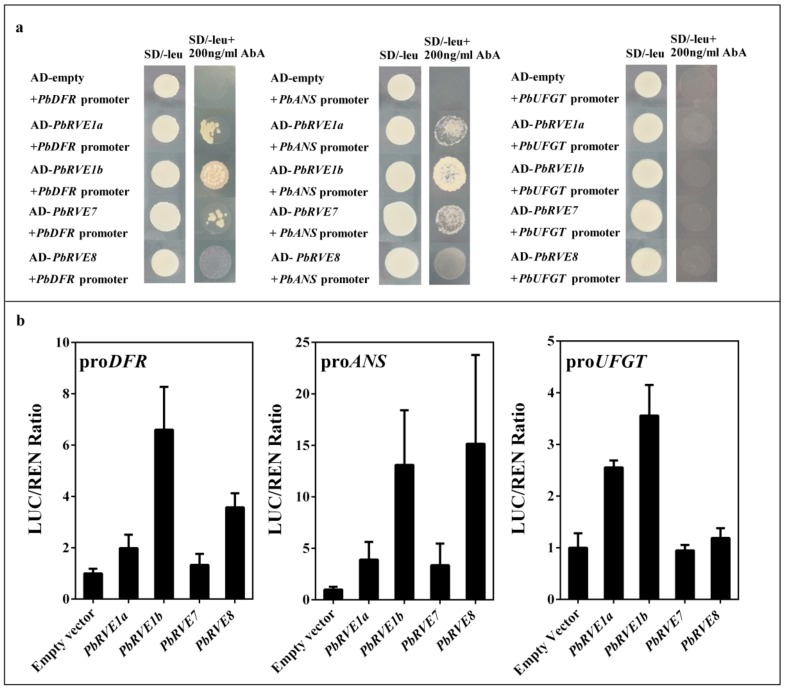
*ALBG* promoters are bound and stimulated by *PbRVE*s. (**a**) Yeast one-hybrid assays of *PbRVE*s with the *PbDFR*, *PbANS* and *PbUFGT* promoters. (**b**) Validation of the activation effects of *PbRVE*s on the *PbDFR*, *PbANS* and *PbUFGT* promoters using a dual-luciferase assay in *Nicotiana benthamiana* leaves. The relative promoter activity is represented by the expression ratio of the structural luciferase (*LUC*) gene to the 35S Renilla (*REN*) gene. The ratio was further standardized based on the LUC/REN value of the empty vector. Results represent the means of five biological replicates. All significant differences are based on comparisons with the control. Error bars show the SEs of the means (*n* = 5).

To determine whether *PbRVE*s regulated *PbDFR*, *PbANS* and *PbUFGT* directly, yeast one-hybrid (Y1H) tests were conducted. *PbRVE1a, 1b,* and *7* bound directly to the *PbDFR* and *PbANS* promoters (Figure 5a). However, direct interactions between the *PbRVE*s and *PbUFGT* were not detected using the Y1H test (Figure 5a). To determine the effects of *PbRVE*s on *PbDFR*, *PbANS* and *PbUFGT*, the promoter regions of *PbDFR*, *PbANS* and *PbUFGT* were used in a dual-luciferase assay system in *Nicotiana benthamiana* leaves. Infiltration with *PbRVE*s activated the promoters of *PbDFR*, *PbANS* and *PbUFGT*, and *PbRVE1b* showed a strongly ability to activate the promoters of *PbDFR*, *PbANS* and *PbUFGT* (Figure 5b). Thus, *PbRVE*s appear to activate directly the promoters of *PbDFR*, *PbANS* and *PbUFGT*, resulting in higher anthocyanin accumulations. 

## 3. Discussion

### 3.1. PbRVE1a, 1b, 7 and 8 Expression Levels Correlated with Anthocyanin Accumulation during the Nighttime in ‘Red Zaosu’ Pear Fruit Skin

The typical anthocyanin biosynthesis-regulating TFs, such as *MYB10* and *HY5*, play important roles in the anthocyanin biosynthetic pathways of fruit [1,21,28]. In *A. thaliana*, *HY5* promotes anthocyanin biosynthesis by binding directly to the promoter regions of *ALBG*s, such as *DFR*, *LDOX* and *UF3GT* [1]. *MYB10* positively activates *DFR* in the anthocyanin biosynthesis of apple [28,29]. *MYB10* and *MYB10b* have positive functions in regulating anthocyanin biosynthesis and accumulation in pear [20,30,31]. However, daily fluctuations in expression were not observed for the typical anthocyanin-related TFs involved in anthocyanin biosynthesis (Figure 2). Therefore, these typical anthocyanin-related TFs are not the main elements active in the anthocyanin biosynthesis pathway during the nighttime in ‘Red Zaosu’ fruit skin. Consequently, in this study, we investigated the TFs involved in the overnight accumulation of anthocyanins in ‘Red Zaosu’ fruit skin.

The single MYB-like TF, *AtRVE8*, has been identified as an activator in the anthocyanin biosynthetic pathway of *A. thaliana* [27]. Based on the phylogenetic tree and expression patterns analysis between *PbRVE*s and *AtRVE*s, *PbRVE1a*, *1b*, *7* and *8* were selected for further investigation (Figure 3a,b). The expression patterns of *PbRVE1a*, *1b*, *7* and *8* showed diurnal oscillations and increased during the nighttime in ‘Red Zaosu’ fruit (Figure 3c). In other red pears, anthocyanin did not accumulate in the daytime (Supplementary Figure S1a). Moreover, the expression patterns of *PbRVE1a*, *1b*, *7* and *8* peaked near dawn in red pear fruit skin (Figure 3c, Supplementary Figure S1b). This result was consistent with the occurrence of anthocyanin accumulation during the nighttime in ‘Red Zaosu’ pear fruit skin (Figure 1a). The data indicate that *PbRVE1a, 1b, 7*, and *8* are potential TFs involved in the nighttime increase in anthocyanin accumulation in ‘Red Zaosu’ pear fruit.

### 3.2. PbRVEs Promoted Anthocyanin Accumulation by Up-Regulating the Expression Levels of PbDFR and PbANS in Pear Fruit Skin

*ALBG*s (such as *DFR*, *ANS* and *UFGT*) are involved in the anthocyanin biosynthetic pathways of fruits [13]. The expression levels of anthocyanin-related structural genes exhibit diurnal oscillation patterns and appear to be regulated by the circadian clock in *A. thaliana* [24]. Additionally, in *A. thaliana*, the expression levels of the anthocyanin-related genes appear to change during light/dark cycles [27]. *AtRVE8* up-regulates the expression of anthocyanin biosynthetic genes in *RVE8*-OE *A. thaliana* plants [27]. In this study, the expression levels of *PbDFR*, *PbANS* and *PbUFGT* exhibited diurnal oscillations and increased during the night in ‘Red Zaosu’ pear fruit skin (Figure 1b). The transient over-expression assay showed that the *PbRVE*s had different abilities to up-regulate the expression levels of structural genes (Figure 4). According to the transient overexpression assay, *PbRVE1b* had a stronger ability than the other *RVE*s to increase anthocyanin biosynthesis in pear fruitlet skins (Figure 4a,b). Furthermore, transient VIGS assays indicated that anthocyanin did not accumulate when *PbRVE1b* was silenced in ‘Palacer’ pear fruitlet skin (Supplementary Figure S3).

In *A. thaliana*, *RVE8* directly binds and regulates the expression of anthocyanin structural gene promoters in response to the diurnal oscillation in anthocyanin accumulation [27,32]. However, the Y1H assay verified that *PbRVE1a*, *1b* and *7*, but not *PbRVE8*, bound directly to the promoters of *PbDFR* and *PbANS* (Figure 5a). The function of *PbRVE8* in binding to the promoters of *PbDFR* and *PbANS* in pear has been precluded by other proteins [27]. In this study, the correlation analysis indicated that the expression levels of *PbRVE*s were significantly positively correlated with the expression levels of *PbDFR*, *PbANS* and *PbUFGT* (Table 1). Thus, we inferred that *PbDFR* and *PbANS* are directly downstream factors of *PbRVE1a*, *1b* and *7* in ‘Red Zaosu’ pear.

Using the dual-luciferase assay, we determined that the expression of *PbRVE1b* increases the activities of the *PbDFR*, *PbANS* and *PbUFGT* promoters (Figure 5b). However, *PbRVE1a* and *PbRVE1b* did not bind directly with the *PbUFGT* promoter (Figure 5a). This result was consistent with the transient overexpression of the *PbRVE*s (Figure 4c). We speculated that *PbRVE1a* and *PbRVE1b* do not directly affect the *PbUFGT* promoter in pear. In *N. benthamiana* leaves, *RVE1a* and *RVE1b* may interact with other factors to active the *UFGT* promoter [33,34,35]. Thus, *PbRVE1a*, *1b* and *7* increase anthocyanin accumulation by directly binding and activating *PbDFR* and *PbANS* in pear fruit.

## 4. Materials and Methods

### 4.1. Plant Material and Treatments

The ‘Red Zaosu’ pear (*P. bretschneideri* Rehd.) is a bud sport of ‘Zaosu’ pear and has characteristic red fruit and leaves. The regulatory mechanism of anthocyanin biosynthesis in ‘Red Zaosu’ has been studied [20,36]. Therefore, we chose ‘Red Zaosu’ to investigate the diurnal accumulation of anthocyanin.

The fruit of ‘Red Zaosu’ was selected from a commercial orchard in Mei County, Shaanxi Province, China, in 2018. The fruit of ‘Red Zaosu’ and ‘Palacer’ (*P. communis* L.) were selected at approximately 40 d after flower blossom and bagged for 30 d until the red pigments totally faded. Then, the fruit of ‘Red Zaosu’ was exposed to daylight for three continuous days. The experiment was conducted on 12–14 June 2018. Additionally, the fruit of ‘Red Zaosu’ were harvested at 0, 3, 6, 9, 12, 24, 27, 30, 33, 36, 48, 50, 54, 57 and 60 hours after sunrise of day 1 (HAS). The faded ‘Palacer’ fruitlets were used for the *PbRVE* virus-induced gene silencing (VIGS) assay. The skins of these harvested fruit were frozen in liquid nitrogen and stored at −80 °C for the subsequent measurement of the anthocyanin content and RNA extraction.

For the dual-luciferase assay infiltration, *N. benthamiana* seedlings were grown in a light incubator (16-h light/8-h dark) at 22 °C.

### 4.2. Anthocyanin Content Measurements

The pH differential method was used to measure the total anthocyanin contents of red skin pear fruitlets [37]. The extraction of total anthocyanins was performed using a previously reported method, with slight modifications [38,39]. Approximately 0.2 g samples of fruit skin were powdered in liquid nitrogen and mixed with PVP-K30 (Sigma, St. Louis, MI, USA), and then 1.5 mL of 1% HCL–methanol was added to the mixed sample. After centrifugation at 4 °C and 12, 000× *g* for 5 min, 200-µL aliquots of the supernatant were transferred separately to two clear tubes for dilution. One was diluted with 400 µL 0.025 M potassium chloride buffer (pH 1.0), and the other with 400 µL 0.4 M sodium acetate buffer (pH 4.5). These solutions were placed for 15 min in the dark at room temperature before the absorbance values were measured synchronously at 520 nm and 700 nm using a Microporous plate spectrophotometer (Multiskan GO; Thermo Scientific, Waltham, MA, USA).

### 4.3. Isolation of RVE Genes and Their Phylogenetic Analysis

The sequences of selected pear *RVE*s were isolated from pear databases [22] (http://peargenome.njau.edu.cn/, access date 1 March 2018). The *RVE*s from pear and *A. thaliana* were aligned using ClustalW (MEGA 7.0, The Biodesign Institute, Arizona State University, AZ, USA) [20]. The phylogenetic analysis was performed with the Minimum-Evolution method and the JTT model using the MEGA 7.0 program (The Biodesign Institute, Arizona State University, AZ, USA) [20]. The GenBank accession numbers for the functionally labelled *RVE*s are listed in Supplementary Table S1. The complete coding DNA sequences (CDSs) of candidate *RVE*s were cloned using PrimeSTAR Max Premix (TaKaRa, Dalian, China) and gene-specific primers (Supplementary Table S2) from ‘Red Zaosu’ cDNA sources.

### 4.4. RNA Isolation and an Expression Analysis Using Quantitative Real-Time PCR (qRT-PCR)

The total RNA of skins was extracted using the RNA prep Pure Plant Kit (Tiangen, Beijing, China). The RNA concentration and quality were detected by UV spectrophotometry and a 0.8% agar gel. In total, 1 µg of total RNA was reverse-transcribed to cDNA using the PrimeScript RT reagent kit with gDNA Eraser (TaKaRa, Dalian, China). The primers used for qRT-PCR were designed with Oligo 7 software (Molecular Biology Insights, Inc., Colorado Springs, USA) and synthesized by AuGCT Biotechnology Synthesis Lab (Beijing, China). The qRT-PCR was performed on an Applied Biosystems StepOnePlus™ Real-Time PCR Systems (Applied Biosystems, Waltham, MA, USA) with TB Green Premix Ex Taq II (Tli RNaseH Plus; TaKaRa, Dalian, China) according to the manufacturer’s instructions. Data were analyzed using the 2^−ΔΔCT^ method. All the qRT-PCR reactions were replicated three times for each biological repeat. The primers for actin, anthocyanin biosynthetic genes and candidate *RVE*s are listed in Supplementary Table S2.

### 4.5. Transient Expression Assay in Pear Fruitlet Skins

The complete CDSs of RVE TFs were cloned into the multiple cloning site (MCS) (BamHI–HindIII) of the pGreenII 0029 62-SK binary vector to form *PbRVE*-OE plasmids (*PbRVE1a*-OE, *PbRVE1b*-OE, *PbRVE7*-OE and *PbRVE8*-OE) [40]. The complete GUS CDS in the pBI121-GUS plasmid was cloned into the MCS of the pGreenII 0029 62-SK binary vector to form the pGreenII 0029 62-SK-GUS plasmid (described in Supplementary Figure S4a). The 400–600-bp fragments of the *C*-termini of RVE TFs were inserted into the MCS (BamHI–XhoI) of pTRV2 to form *PbRVE* VIGS vectors (*PbRVE1a*-TRV, *PbRVE1b*-TRV, *PbRVE7*-TRV and *PbRVE8*-TRV, described in Supplementary Figure S4b). The primers for amplifying the sequences are described in Supplementary Table S2. *Agrobacterium tumefaciens* strain EHA105 independently containing the constructed plasmids was grown at 28 °C on Luria–Bertani (LB) solid medium supplemented with 50 mg/L kanamycin and 25 mg/L rifampicin. After incubating for 48 h, the *A. tumefaciens* was resuspended in the infiltration buffer (10 mM MgCl_2_, 10 mM MES and 200 µM acetosyringone) and shaken for 3–4 h (up to a final OD600 of 0.8) at room temperature before being injected into pear fruitlet skins. The pear injection process was as described in Spolaore et al. [41] and the injection volume was as described in Zhai et al. [20]. The negative controls were infiltrated with *A. tumefaciens* containing pGreenII 0029 62-SK or the pTRV2 empty vector. The treated fruitlets were harvested at 5 d after injection. For GUS staining, the plant materials were stained with 5-bromo-4-chloro-3-indolyl glucuronide at 37 °C for 12 h as described previously [42].

### 4.6. Y1H Assay

The Y1H assays were performed following the manufacturer’s instructions for the Matchmaker Gold Yeast One-Hybrid System Kit (Clontech, Mountain View, CA, USA). We ligated independently ~800-bp fragments of the *PbANS* and *PbDFR* promoters into pAbAi to construct the pAbAi-baits. Additionally, the complete CDSs of the *PbRVE*s were separately inserted into the pGADT7 vector to construct the prey-AD vectors. The pAbAi-bait vectors were linearized and transformed into Y1HGold separately. The colonies were selected in the absence of uracil on selective medium-containing agar plates. The correct integration of plasmids into the genome of the Y1HGold yeast was confirmed using a colony PCR analysis (Matchmaker Insert Check PCR Mix 1; Clontech, Mountain View, CA, USA). After determining the minimal inhibitory concentration of Aureobasidin A (AbA) for the bait–reporter yeast strains, the AD-prey vectors were transformed into the bait yeast strains and selected on synthetic dextrose (SD)/−Leu/AbA plates. All the transformations and screenings were performed three times.

### 4.7. Dual-Luciferase Assay

The promoters of *PbANS* and *PbDFR* were amplified from ‘Red Zaosu’ genomic DNA using PrimeSTAR Max Premix (TaKaRa, Dalian, China) and gene-specific primers (Supplementary Table S1). These promoters were cloned into the HindIII and BamHI sites within pGreenII 0800-LUC [40]. The full-length CDS sequences of *PbRVE1a*, *PbRVE1b*, *PbRVE7* and *PbRVE8* were independently cloned into the MCS (BamHI-HindIII) of the pGreenII 0029 62-SK binary vector [40].

Each of these recombinant plasmids and the pSoup helper plasmid were transferred individually into *A. tumefaciens* strain EHA105 [40]. The *A. tumefaciens* cells containing *PbRVE*-SKs were separately mixed with pro*PbDFR*-LUC or pro*PbANS*-LUC at 1:1 ratio before being infiltrated into 4-week-old *N. benthamiana* leaves. After injection, the plants were grown for 3 d in a light incubator (16-h light/8-h dark) at 22 °C, and then, the treated leaves were collected in 1 × phosphate buffered solution for the dual-luciferase assay. The Firefly luciferase (Luc) to Renilla luciferase (Ren) activity ratios were analyzed using a Dual-Luciferase^®^ Reporter Assay System (Promega, Madison, WI, USA) on a Tecan Infinite M200pro Full-Wavelength Multifunctional Enzyme Labelling Instrument (TECAN, Männedorf, Switzerland). Three independent experiments were carried out with at least five biological replicates per experiment.

### 4.8. Statistical Analysis

All the experimental data were statistically processed using GraphPad Prism 6 software (GraphPad Software Inc., San Diego, CA, USA) and are shown as means ± standard errors (SEs). Additionally, the significant differences were analyzed using a one-way analysis of variance and Student’s *t*-tests. 

## Figures and Tables

**Table 1:** Correlation analysis between the expression of anthocyanin-related transcription factors (TFs) and the expression of *ALBG*s in pear fruit.

Correlation Coefficients (Pearson’s)								
Genes	*PbMYB9*	*PbMYB10*	*PbMYB10b*	*PbbHLH33a*	*PbRVE1a*	*PbRVE1b*	*PbRVE7*	*PbRVE8*
*PbANS*	0.441 **	0.22	−0.01	0.07	0.750 **	0.775 **	0.728 **	0.757 **
*PbDFR*	0.416 **	0.17	−0.02	0.08	0.734 **	0.782 **	0.746 **	0.777 **
*PbUFGT*	0.355 *	0.335 *	0.19	0.06	0.779 **	0.859 **	0.818 **	0.855 **

The pairwise correlation coefficients between the expression levels of anthocyanin-related TFs and those of *ALBG*s in the fruit skin of ‘Red Zaosu’ pear over three continuous days. The NCBI accessions of the anthocyanin-related genes are listed in Supplementary Table S1. The significant correlation coefficients are indicated in bolded values. The data was analyzed using SPSS 20. *: Correlation significant at the 0.05 level (*p *< 0.05, two-tailed); **: Correlation significant at the 0.01 level (*p *< 0.01, two-tailed).

## Supplementary Materials

Supplementary materials can be found at https://www.mdpi.com/1422-0067/21/5/1634/s1. Figure S1: The anthocyanin content of red pear fruit and expression patterns of candidate *PbRVEs* in red pear fruit; Figure S2: The GUS-stained ‘Zaosu’ fruitlets skin infiltrated by pGreen II 62-SK-GUS; Figure S3: Functional analysis of the *PbRVEs* using VIGS in pear fruitlets skin; Figure S4: Construction of the recombinant plasmid; Table S1: The accession numbers of the genes used in this study; Table S2: List of primers used in this study; Table S3: The actual transcript abundance data of *PbRVEs*.

## Abbreviations

ANS	Anthocyanidin synthase
ALBGs	Anthocyanin late biosynthetic genes
CDS	Coding DNA sequence
DFR	Dihydroflavonol 4-reductase
GUS	the β-glucuronidase gene
HAS	Hours after sunrise of day 1
LUC	Firefly luciferase
MCS	Multiple cloning sites
MBW	MYB–bHLH–WDR
OE	Overexpression
qRT-PCR	Quantitative Real-Time PCR
REN	Renilla luciferase
RVE	REVEILLE
SD	Selective synthetic dextrose medium
SEs	the standard errors
TF	Transcription factor
UFGT	UDP-glucoside: flavonoid glucosyltransferase
VIGS	Virus-induced gene silencing
Y1H	Yeast one-hybrid assay

## References

[1] Shin D.H., Choi M., Kim K., Bang G., Cho M., Choi S.B., Choi G., Park Y.I. (2013). HY5 regulates anthocyanin biosynthesis by inducing the transcriptional activation of the MYB75/PAP1 transcription factor in *Arabidopsis*. FEBS Lett..

[2] Liu Y., Tikunov Y., Schouten R.E., Marcelis L.F.M., Visser R.G.F., Bovy A. (2018). Anthocyanin Biosynthesis and Degradation Mechanisms in *Solanaceous* Vegetables: A Review. Front. Chem..

[3] Sarma A.D., Sharma R. (1999). Anthocyanin-DNA copigmentation complex: Mutual protection against oxidative damage. Phytochemistry.

[4] Tanaka Y., Sasaki N., Ohmiya A. (2008). Biosynthesis of plant pigments: Anthocyanins, betalains and carotenoids. Plant J..

[5] Shang Y., Venail J., Mackay S., Bailey P.C., Schwinn K.E., Jameson P.E., Martin C.R., Davies K.M. (2011). The molecular basis for venation patterning of pigmentation and its effect on pollinator attraction in flowers of *Antirrhinum*. New Phytol..

[6] Petroni K., Pilu R., Tonelli C. (2014). Anthocyanins in corn: A wealth of genes for human health. Planta.

[7] Li D., Wang P., Luo Y., Zhao M., Chen F. (2015). Health benefits of anthocyanins and molecular mechanisms: Update from recent decade. Crit. Rev. Food Sci. Nutr..

[8] Wang L.S., Stoner G.D. (2008). Anthocyanins and their role in cancer prevention. Cancer Lett..

[9] De Pascual-Teresa S. (2014). Molecular mechanisms involved in the cardiovascular and neuroprotective effects of anthocyanins. Arch. Biochem. Biophys..

[10] Sakuta M. (2013). Diversity in plant red pigments: Anthocyanins and betacyanins. Plant Biotechnol. Rep..

[11] Xu W., Dubos C., Lepiniec L. (2015). Transcriptional control of flavonoid biosynthesis by MYB-bHLH-WDR complexes. Trends Plant Sci..

[12] Lin-Wang K., Micheletti D., Palmer J., Volz R., Lozano L., Espley R., Hellens R.P., Chagne D., Rowan D.D., Troggio M. (2011). High temperature reduces apple fruit colour via modulation of the anthocyanin regulatory complex. Plant Cell Environ..

[13] Jaakola L. (2013). New insights into the regulation of anthocyanin biosynthesis in fruits. Trends Plant Sci..

[14] Xu W., Grain D., Bobet S., Le Gourrierec J., Thevenin J., Kelemen Z., Lepiniec L., Dubos C. (2014). Complexity and robustness of the flavonoid transcriptional regulatory network revealed by comprehensive analyses of MYB-bHLH-WDR complexes and their targets in *Arabidopsis* seed. New Phytol..

[15] Katia P., Chiara T. (2011). Recent advances on the regulation of anthocyanin synthesis in reproductive organs. Plant Sci..

[16] Gonzalez A., Zhao M., Leavitt J.M., Lloyd A.M. (2008). Regulation of the anthocyanin biosynthetic pathway by the TTG1/bHLH/Myb transcriptional complex in *Arabidopsis* seedlings. Plant J..

[17] Maier A., Schrader A., Kokkelink L., Falke C., Welter B., Iniesto E., Rubio V., Uhrig J.F., Hulskamp M., Hoecker U. (2013). Light and the E3 ubiquitin ligase COP1/SPA control the protein stability of the MYB transcription factors PAP1 and PAP2 involved in anthocyanin accumulation in *Arabidopsis*. Plant J..

[18] Xie X.B., Li S., Zhang R.F., Zhao J., Chen Y.C., Zhao Q., Yao Y.X., You C.X., Zhang X.S., Hao Y.J. (2012). The bHLH transcription factor MdbHLH3 promotes anthocyanin accumulation and fruit colouration in response to low temperature in apples. Plant Cell Environ..

[19] Xie Y., Chen P., Yan Y., Bao C., Li X., Wang L., Shen X., Li H., Liu X., Niu C. (2018). An atypical R2R3 MYB transcription factor increases cold hardiness by CBF-dependent and CBF-independent pathways in apple. New Phytol..

[20] Zhai R., Wang Z., Zhang S., Meng G., Song L., Wang Z., Li P., Ma F., Xu L. (2016). Two MYB transcription factors regulate flavonoid biosynthesis in pear fruit (*Pyrus bretschneideri* Rehd.). J. Exp. Bot..

[21] Feng S., Wang Y., Yang S., Xu Y., Chen X. (2010). Anthocyanin biosynthesis in pears is regulated by a R2R3-MYB transcription factor PyMYB10. Planta.

[22] Wu J., Wang Z., Shi Z., Zhang S., Ming R., Zhu S., Khan M.A., Tao S., Korban S.S., Wang H. (2013). The genome of the pear (*Pyrus bretschneideri* Rehd.). Genome Res..

[23] Harmer S.L. (2009). The circadian system in higher plants. Annu. Rev. Plant Biol..

[24] Deikman J., Hammer P.E. (1995). Induction of Anthocyanin Accumulation by Cytokinins in *Arabidopsis thaliana*. Plant Physiol..

[25] Dubos C., Le Gourrierec J., Baudry A., Huep G., Lanet E., Debeaujon I., Routaboul J.M., Alboresi A., Weisshaar B., Lepiniec L. (2008). MYBL2 is a new regulator of flavonoid biosynthesis in *Arabidopsis thaliana*. Plant J..

[26] Nguyen N.H., Jeong C.Y., Kang G.H., Yoo S.D., Hong S.W., Lee H. (2015). MYBD employed by HY5 increases anthocyanin accumulation via repression of *MYBL2* in *Arabidopsis*. Plant J..

[27] Perez-Garcia P., Ma Y., Yanovsky M.J., Mas P. (2015). Time-dependent sequestration of RVE8 by LNK proteins shapes the diurnal oscillation of anthocyanin biosynthesis. Proc. Natl. Acad. Sci. USA.

[28] Espley R.V., Hellens R.P., Putterill J., Stevenson D.E., Kutty-Amma S., Allan A.C. (2007). Red colouration in apple fruit is due to the activity of the MYB transcription factor, MdMYB10. Plant J..

[29] Li K.T., Zhang J., Kang Y.H., Chen M.C., Song T.T., Geng H., Tian J., Yao Y.C. (2018). McMYB10 Modulates the Expression of a Ubiquitin Ligase, McCOP1 During Leaf Coloration in Crabapple. Front. Plant Sci..

[30] Bai S., Tao R., Tang Y., Yin L., Ma Y., Ni J., Yan X., Yang Q., Wu Z., Zeng Y. (2019). BBX16, a B-box protein, positively regulates light-induced anthocyanin accumulation by activating *MYB10* in red pear. Plant Biotechnol. J..

[31] Yao G., Ming M., Allan A.C., Gu C., Li L., Wu X., Wang R., Chang Y., Qi K., Zhang S. (2017). Map-based cloning of the pear gene *MYB114* identifies an interaction with other transcription factors to coordinately regulate fruit anthocyanin biosynthesis. Plant J..

[32] Nguyen N.H., Lee H. (2016). MYB-related transcription factors function as regulators of the circadian clock and anthocyanin biosynthesis in *Arabidopsis*. Plant Signal. Behav..

[33] Xing H., Wang P., Cui X., Zhang C., Wang L., Liu X., Yuan L., Li Y., Xie Q., Xu X. (2015). LNK1 and LNK2 recruitment to the evening element require morning expressed circadian related MYB-like transcription factors. Plant Signal. Behav..

[34] Wang Z.-Y., Kenigsbuch D., Sun L., Harel E., Ong M.S., Tobin E.M. (1997). A Myb-related transcription factor is involved in the phytochrome regulation of an *Arabidopsis Lhcb* gene. Plant Cell.

[35] Andronis C., Barak S., Knowles S.M., Sugano S., Tobin E.M. (2008). The clock protein CCA1 and the bZIP transcription factor HY5 physically interact to regulate gene expression in *Arabidopsis*. Mol. Plant.

[36] Zhai R., Liu X.T., Feng W.T., Chen S.S., Xu L.F., Wang Z.G., Zhang J.L., Li P.M., Ma F.W. (2014). Different biosynthesis patterns among flavonoid 3-glycosides with distinct effects on accumulation of other flavonoid metabolites in pears (*Pyrus bretschneideri* Rehd.). PLoS ONE..

[37] Wolfe K.L., Liu R.H. (2003). Apple peels as a value-added food ingredient. J. Agric. Food Chem..

[38] Giusti M.M., Wrolstad R.E. (2001). Characterization and Measurement of Anthocyanins by UV-Visible Spectroscopy. Curr. Protoc. Food Anal. Chem..

[39] Wang Z., Du H., Zhai R., Song L., Ma F., Xu L. (2017). Transcriptome Analysis Reveals Candidate Genes Related to Color Fading of ‘Red Bartlett’ (*Pyrus communis* L.). Front. Plant Sci..

[40] Hellens R.P., Allan A.C., Friel E.N., Bolitho K., Grafton K., Templeton M.D., Karunairetnam S., Gleave A.P., Laing W.A. (2005). Transient expression vectors for functional genomics, quantification of promoter activity and RNA silencing in plants. Plant Methods.

[41] Spolaore S., Trainotti L., Casadoro G. (2001). A simple protocol for transient gene expression in ripe fleshy fruit mediated by *Agrobacterium*. J. Exp. Bot..

[42] Fillatti J.J., Kiser J., Rose R., Comai L. (1987). Efficient transfer of a glyphosate tolerance gene into tomato using a binary *Agrobacterium tumefaciens* vector. Biotechnology.

