# Supplementary material for: REVEILLE Transcription Factors Contribute to the Nighttime Accumulation of Anthocyanins in ‘Red Zaosu’ (Pyrus Bretschneideri Rehd.) Pear Fruit Skin
Source: Int J Mol Sci. 2020 Feb 27;21(5):1634. doi: 10.3390/ijms21051634 (PMC7084243; doi:10.3390/ijms21051634)
Supplement: Supplementary file 1 [file ijms-21-01634-s001.zip › Supplementary material/EdanzEditingCertificate-77448.pdf]

理文编辑

Certificate of Editing

Edited provisional title

REVEILLE transcription factors contribute to the nighttime accumulation of anthocyanins in ‘Red Zaosu’ (Pyrus bretschneideri Rehd.) pear fruit skin

Client name and institution

Xieyu Li , College of Horticulture, Northwest A&F University, Taicheng Road NO.3, Yangling, Shaanxi Province, China

Date Completed

Identification code
77448

Certificate issued by
Koji Yamashita
Managing Director and CEO

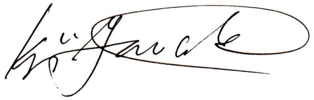

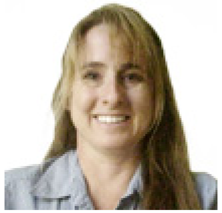

Expert Editor: Lesley Benyon
1999 PhD Genetics and Molecular Biology
University of North Carolina
Biochemistry and Cell Biology, Genetics, Plant Biology

www.liwenbianji.cn
While this certificate confirms the authors have used Edanz's editing services, we cannot guarantee that additional changes have not been made after our edits.
