# Supplementary material for: REVEILLE Transcription Factors Contribute to the Nighttime Accumulation of Anthocyanins in ‘Red Zaosu’ (Pyrus Bretschneideri Rehd.) Pear Fruit Skin
Source: Int J Mol Sci. 2020 Feb 27;21(5):1634. doi: 10.3390/ijms21051634 (PMC7084243; doi:10.3390/ijms21051634)
Supplement: Supplementary file 1 [file ijms-21-01634-s001.zip › Supplementary material/Supplementary Figures.pdf]

Figure S1

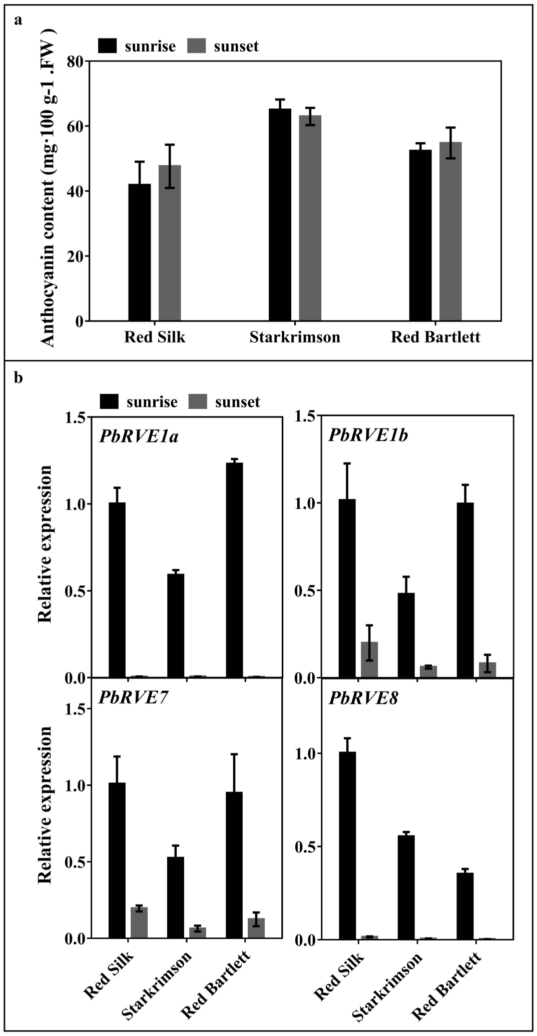

The anthocyanin content of red pear fruit and expression patterns of candidate *PbRVEs* in red pear fruit.

a the anthocyanin content in the skin of 'Red Silk', 'Starkrimson' and 'Red Bartlett' fruit at the sunrise (0 HAS) and the sunset (12 HAS) in the same day.

b The expression patterns of *PbRVE1a*, *1b*, *7* and *8* in the skin of 'Red Silk', 'Starkrimson' and 'Red Bartlett' fruit at the sunrise (0 HAS) and the sunset (12 HAS) in the same day.

The black boxes mean the sunrise; the gray boxes mean the sunset. The NCBI accession numbers of the used typical transcription factors are listed in Supplementary File 1: Table S1. The gene accession numbers used are listed in Supplementary File 1: Table S2. Error bars represent standard errors of the means (SEs; n = 3).

Figure S2

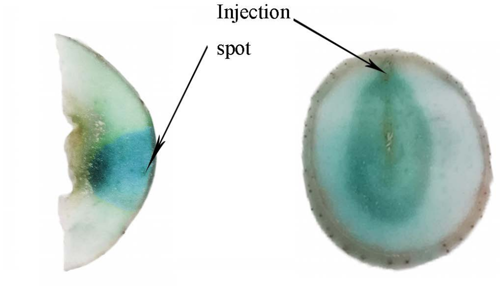

The GUS-stained ‘Zaosu’ fruitlets skin infiltrated by pGreen II 62-SK-GUS. GUS reporter was applied to identify the gene expression pattern in the infected fruitlets skin. The blue region indicates that the *GUS* gene expressed in this area which was consisted with the observation of the expression patterns of *PbRVEs* over-expressed ‘Zaosu’ fruitlets skin.

Figure S3

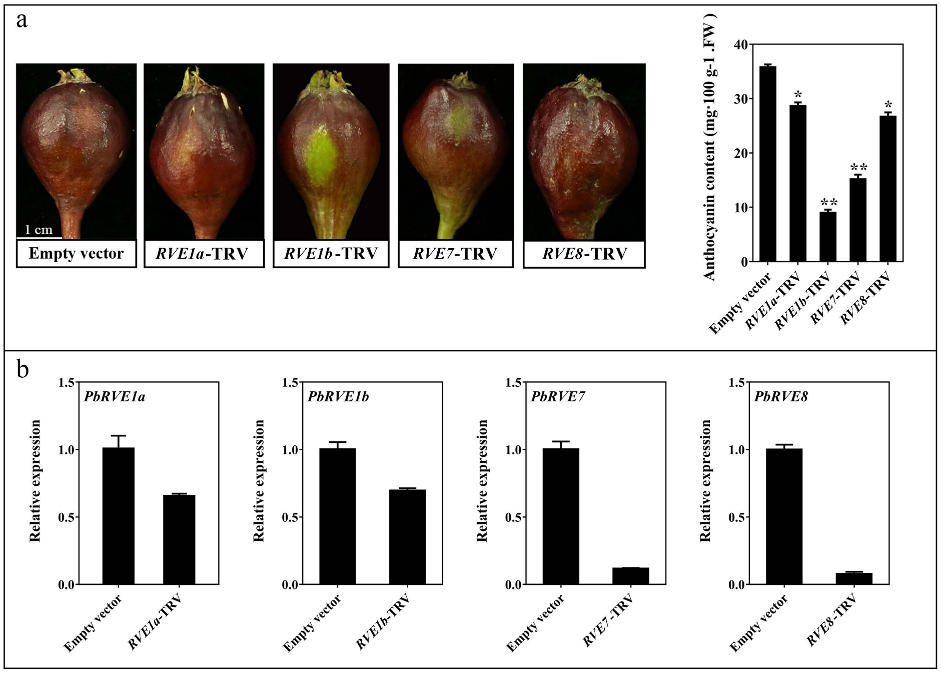

Functional analysis of the *PbRVEs* using VIGS in pear fruitlets skin.

a Transient VIGS assays and anthocyanin contents of *RVE1a*-TRV, *RVE1b*-TRV, *RVE7*-TRV and *RVE8*-TRV in Palacer fruitlets skin.

b The expression levels of *PbRVEs* in *RVE1a*-TRV, *RVE1b*-TRV, *RVE7*-TRV and *RVE8*-TRV fruitlets skin.

All of the significant differences are based on comparisons with the control. Error bars represent standard errors of the means (SEs; n = 3). Data in (a) was analyzed with Student's *t*-test: $*P < 0.05$, $**P < 0.01$.

Figure S4

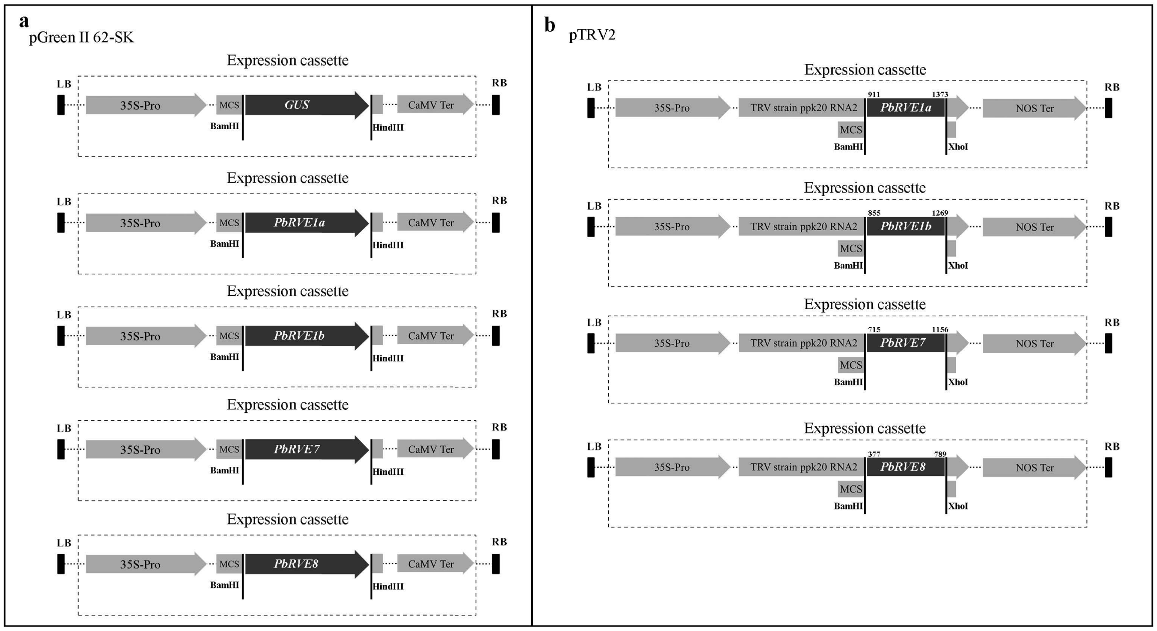

Construction of the recombinant plasmid.

a Structural of the recombinant plasmid of pGreen II 62-SK with the CDS of *GUS* and *PbRVEs*. The dark gray arrows indicated the CDS of *GUS*, *PbRVE1a*, *1b*, *7* and *8*. 35S-pro: CaMV 35S promoter, CaMV Ter: CaMV terminator, MCS: multiple cloning site, LB: left T-DNA border, RB: right T-DNA border.

b Structural of the recombinant plasmid of pTRV2 with the fragments of PbRVEs. The dark gray boxes indicated the fragments of *PbRVE1a*, *1b*, *7* and *8*. The numbers upon the dark gray boxes indicated that the start and end sites of the fragments in CDS of *PbRVE1a*, *1b*, *7* and *8*. 35S-pro: CaMV 35S promoter, MCS: multiple cloning site, NOS Ter: NOS terminator, LB: left T-DNA border, RB: right T-DNA border.

The dashed boxes in (a) and (b) indicate the expression cassettes of vectors.
